# Quantum Dot Passivation of Halide Perovskite Films with Reduced Defects, Suppressed Phase Segregation, and Enhanced Stability

**DOI:** 10.1002/advs.202102258

**Published:** 2021-11-29

**Authors:** Long Hu, Leiping Duan, Yuchen Yao, Weijian Chen, Zizhen Zhou, Claudio Cazorla, Chun‐Ho Lin, Xinwei Guan, Xun Geng, Fei Wang, Tao Wan, Shuying Wu, Soshan Cheong, Richard D. Tilley, Shanqin Liu, Jianyu Yuan, Dewei Chu, Tom Wu, Shujuan Huang

**Affiliations:** ^1^ School of Engineering Macquarie University Sustainable Energy Research Centre Macquarie University Sydney NSW 2109 Australia; ^2^ School of Materials Science and Engineering University of New South Wales (UNSW) Sydney NSW 2052 Australia; ^3^ School of Photovoltaic and Renewable Energy Engineering University of New South Wales (UNSW) Sydney NSW 2052 Australia; ^4^ Departament de Física Universitat Politècnica de Catalunya Campus Nord B4‐B5 Barcelona E‐08034 Spain; ^5^ Electron Microscope Unit Mark Wainwright Analytical Centre University of New South Wales (UNSW) Sydney NSW 2052 Australia; ^6^ School of Chemistry and Chemical Engineering Henan Institute of Science and Technology Xinxiang Henan 453003 P. R. China; ^7^ Institute of Functional Nano & Soft Materials (FUNSOM) Jiangsu Key Laboratory for Carbon‐Based Functional Materials and Devices Joint International Research Laboratory of Carbon‐Based Functional Materials and Devices Soochow University 199 Ren‐Ai Road, Suzhou Industrial Park Suzhou Jiangsu 215123 P. R. China

**Keywords:** defect, halide perovskites, phase segregation, quantum dots, solar cells

## Abstract

Structural defects are ubiquitous for polycrystalline perovskite films, compromising device performance and stability. Herein, a universal method is developed to overcome this issue by incorporating halide perovskite quantum dots (QDs) into perovskite polycrystalline films. CsPbBr_3_ QDs are deposited on four types of halide perovskite films (CsPbBr_3_, CsPbIBr_2_, CsPbBrI_2_, and MAPbI_3_) and the interactions are triggered by annealing. The ions in the CsPbBr_3_ QDs are released into the thin films to passivate defects, and concurrently the hydrophobic ligands of QDs self‐assemble on the film surfaces and grain boundaries to reduce the defect density and enhance the film stability. For all QD‐treated films, PL emission intensity and carrier lifetime are significantly improved, and surface morphology and composition uniformity are also optimized. Furthermore, after the QD treatment, light‐induced phase segregation and degradation in mixed‐halide perovskite films are suppressed, and the efficiency of mixed‐halide CsPbIBr_2_ solar cells is remarkably improved to over 11% from 8.7%. Overall, this work provides a general approach to achieving high‐quality halide perovskite films with suppressed phase segregation, reduced defects, and enhanced stability for optoelectronic applications.

## Graphical abstract

A universal approach is reported to fabricate perovskite films by incorporating inorganic CsPbBr_3_ quantum dots (QDs) into halide perovskite bulk films. Upon post‐annealing, the released elements from QDs compensate vacancies, and hydrophobic ligands on QDs passivate under‐charged Pb atoms and self‐assemble on surface. Therefore, the resulting films with reduced trap density, suppressed phase segregation, improved surface uniformity and enhanced stability are enabled.

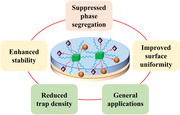

## 1 Introduction

Metal halide perovskites have been widely applied in many kinds of optoelectronic devices such as photovoltaics,^[^ ^1^, ^2^, ^3^ ^]^ photodetectors,^[^ ^4^, ^5^, ^6^ ^]^ light‐emitting diodes,^[^ ^7^, ^8^, ^9^ ^]^ and lasers.^[^ ^10^ ^]^ In the past decade, numerous efforts have been dedicated to optimizing the film quality of halide perovskites. Driven by these efforts, the power conversion efficiency (PCE) of halide perovskite solar cell (PSC) has been boosted to 25.5%, which is on par with silicon‐based photovoltaics, demonstrating great potential in revolutionary renewable energy applications.^[^ ^11^, ^12^ ^]^ The tremendous success of PSCs is attributed to their excellent optoelectronic properties, such as a high absorption coefficient, high charge mobility, long carrier lifetime, tunable bandgap, and small exciton binding energy.^[^ ^13^, ^14^, ^15^, ^16^, ^17^ ^]^ Moreover, the optimization techniques of perovskite absorbing layers were rapidly developed to tap the potential of these emerging materials, including composition engineering,^[^ ^18^ ^]^ heteroatomic doping,^[^ ^19^, ^20^, ^21^ ^]^ surface and interfacial passivation,^[^ ^22^, ^23^, ^24^ ^]^ heterojunction engineering,^[^ ^25^ ^]^ and additive introduction.^[^ ^26^ ^]^ However, it is widely recognized that defect formation is inevitable on surfaces, interfaces, and grain boundaries of perovskite polycrystalline films, which can lead to recombination of photogenerated carriers and suppress the quasi‐Fermi splitting. Additionally, surface and interfacial defects can initiate and accelerate perovskite degradation by generating ion migration channels and thus reduce device stability.^[^ ^27^ ^]^ Although halide perovskites are considered as defect‐tolerant semiconductors,^[^ ^28^ ^]^ the existence of defects still severely restricts the performance of PSCs. Therefore, defect management is a crucial issue that needs to be addressed to further improve the performance and stability of PSCs.

Currently, the most efficient PSCs are achieved by engineering the composition of precursor solution, such as cation incorporation, lead substitution, and halide mixing.^[^ ^29^ ^]^ Besides precursor solution engineering, surface and interfacial passivation is highly desirable to further optimize halide perovskites.^[^ ^30^ ^]^ Lin et al. reported that passivation with an effective patterning method could lead to perovskite‐based electronic and optoelectronic devices in a scalable way without compromising their electronic or optical characteristics.^[^ ^16^ ^]^ Very recently, inorganic perovskite quantum dots (QDs) capped with hydrophobic ligands provide a platform for constructing crystalline thin films via annealing, where nanocrystals would mutually merge into large crystalline grains due to Ostwald ripening.^[^ ^31^, ^32^ ^]^ Meanwhile, organic ligands attached to QDs can self‐assemble on interfaces and grain boundaries for defect passivation.^[^ ^33^ ^]^ Among various perovskite QDs, CsPbBr_3_ QDs are widely applied for constructing optoelectronic devices because of their ultra‐high photoluminescence (PL) quantum yield and superior stability.^[^ ^34^, ^35^, ^36^ ^]^ In addition, benefiting from their soft ion lattice, hydrophobic QDs can be well combined into halide perovskite thin film through ion exchange and diffusion.^[^ ^37^, ^38^, ^39^ ^]^

In this work, we developed a universal QD‐based approach to synthesize perovskite films with concurrent merits of reduced defect density, suppressed phase segregation and enhanced stability. Four types of halide perovskite films (i.e., CsPbBr_3_, CsPbIBr_2_, CsPbBrI_2_, and MAPbI_3_) were coated with inorganic CsPbBr_3_ QDs during the anti‐solvent spin‐coating process. Upon post‐annealing, the QD passivators merged into the nanocrystalline films. We found that the PL emission intensity and carrier lifetime were substantially enhanced, accompanying with improved surface morphology and homogenous composition distribution in all QD‐treated films. Furthermore, after the introduction of QDs, light‐induced phase segregation in CsPbIBr_2_ film was significantly suppressed, and the solar cell PCE was remarkably improved from 8.7% to 11.1%. Detailed density functional theory (DFT) analysis and device characterizations revealed that hydrophobic organic ligands from the QDs self‐assemble at the surfaces and grain boundaries to passivate under‐charged Pb^2+^ and halide vacancies, thereby contributing to the improved quality of halide perovskite films.

## 2 Results and Discussion

**Figure 1:**
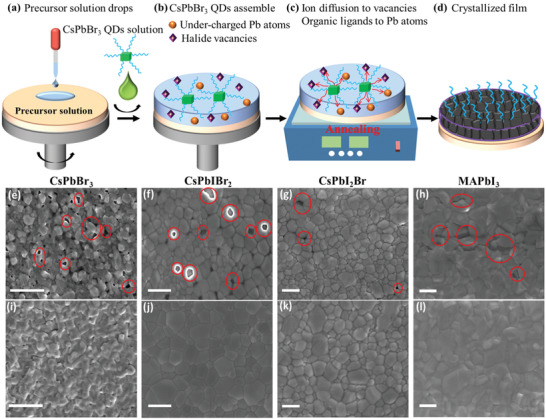
Processing of perovskite films with the QD passivation treatment. a) Precursor solution is spin‐coated on the substrate, then antisolvent containing CsPbBr_3_ QDs is dropped on precursor solution during the spin‐coating process. b) CsPbBr_3_ QDs self‐assemble on the surface of deposited films, which contain halide vacancies and under‐charged Pb. c) The ions released from QDs diffuse into halide vacancies and organic ligands coordinate under‐charged Pb atoms during the annealing. d) The crystallized halide perovskite film. SEM images of four kinds of perovskite films (CsPbBr_3_, CsPbIBr_2_, CsPbI_2_Br, and MAPbI_3_). e–h) without and i–l) with QD treatment. Heterogeneous spots on thin films without the QD treatment are marked with red cycles and ellipses. The scale bar is 300 nm in (e–l).

CsPbBr_3_ QDs were synthesized using a hot‐injection method and purified through precipitation/redispersion according to the reported recipe.^[^ ^36^, ^40^ ^]^ Subsequently, QDs were dispersed into hexane as the anti‐solvent for halide perovskite thin film deposition (details can be found in the Experimental Section). The as‐obtained CsPbBr_3_ QDs were characterized using ultraviolet‐visible (UV‐vis) spectroscopy, steady‐state PL and transmission electron microscopy (TEM) imaging as shown in Figure S1 in the Supporting Information. The whole process of perovskite film deposition with CsPbBr_3_ QD passivation is shown in **Figure** **1**a–d. Halide perovskite precursor in dimethyl formamide (DMF) or dimethyl sulfoxide (DMSO) solution was dropped and spin‐coated on SnO_2_/ITO substrates, followed by the spin‐coating of the anti‐solvent hexane solution containing CsPbBr_3_ QDs with a concentration of 20 mg mL^−1^ to accelerate crystallization. Then, the deposited films treated with QDs were annealed on a hotplate at 150 °C for three kinds of inorganic film, i.e., CsPbBr_3_, CsPbIBr_2_, CsPbBrI_2,_ and at 100 °C for MAPbI_3_ film to form uniform and crystallized films. As a reference, the control perovskite films were also fabricated using the identical procedure but without QDs in the anti‐solvent hexane solution.

To verify the universal effectiveness of the QD treatment method, four kinds of perovskite films were prepared, including CsPbBr_3_, CsPbIBr_2_, CsPbBrI_2_, and MAPbI_3_. Surface morphology is a crucial parameter directly linked to the quality of perovskite film.^[^ ^41^, ^42^ ^]^ Typically, constructing a dense and well‐crystallized perovskite film with large grain size is a prerequisite for achieving highly efficient and long‐term stable optoelectronic devices. Figure 1e–l shows the scanning electron microscopy (SEM) images of four compositional perovskite films without and with QD treatment. It is clear that all QD‐treated perovskite films exhibit a uniform and dense morphology with larger grain crystals. In contrast, it is observed that there are a few heterogeneous spots and pinholes for thin films without QD treatment (marked with red cycles in Figure 1e–h), which suggests less uniformity.^[^ ^43^ ^]^

Previous studies have shown that during the antisolvent spin‐coating process, incorporation of SnS and PbS QDs could induce the nucleation of precursor solution and accelerate the growth of perovskite, which is favorable for achieving dense, pinhole‐free and better‐crystalized perovskite films.^[^ ^44^, ^45^ ^]^ In our case, CsPbBr_3_ QDs can accelerate the crystallization as seeds and in situ fill the voids as well as grain boundaries, and as a result, the CsPbBr_3_ QDs will be integrated with perovskite thin films to form uniform and dense films upon annealing.^[^ ^46^ ^]^ As a consequence, the surface morphology of QD‐treated films is significantly improved. The improved surface morphology can reduce defects and suppress degradation,^[^ ^47^, ^48^ ^]^ which will be discussed later. From the SEM results, we can conclude that all films with QD treatment demonstrate better surface morphology and larger grain crystals with homogenous composition distribution.

**Figure 2:**
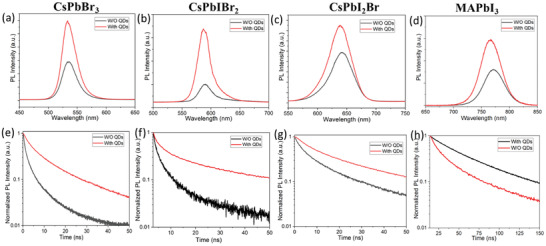
a–d) Steady‐state and e–h) time‐resolved PL measured on four kinds of perovskite films (CsPbBr_3_, CsPbIBr_2_, CsPbI_2_Br, and MAPbI_3_) without and with QD treatment.

PL spectroscopy is a powerful tool to investigate surface trap density.^[^ ^49^ ^]^ Firstly, steady‐state PL measurements were performed on the four kinds of perovskite films as shown in **Figure** **2**a–d. Under laser excitation, perovskite films generate holes and electrons. Subsequently, these photogenerated holes and electrons recombine again, leading to PL emission. Since defect trap states in perovskite films can cause non‐radiative recombination, the stronger PL intensity indicates a lower defect density.^[^ ^50^, ^51^ ^]^ In our PL investigation, all perovskite films with QD treatment demonstrate higher PL intensity compared to their control films, indicating that the perovskite defects are suppressed by QD treatment. Furthermore, carrier dynamics of perovskite films were studied by time‐resolved PL decay as shown in Figure 2e–h. The carrier lifetime in all QD‐treated films is prolonged, indicating that the non‐radiative recombination is reduced. The detailed lifetime was extracted by using single‐exponent fitting as shown in **Table** **1**. The results from steady‐state PL and PL decay confirm that the perovskite defects are reduced after QD treatment, reflecting that this method is universally applicable in all four perovskite compositions studies in this work.

**Table 1:** PL lifetime of four types of perovskite film extracted from the PL decay curves by using single‐exponent fitting

Film Type	CsPbBr_3_	CsPbIBr_2_	CsPbBrI_2_	MAPbI_3_
With QDs	7.90 ns	8.65 ns	9.25 ns	53.2 ns
W/O QDs	2.77 ns	3.81 ns	4.69 ns	23.5 ns

To thoroughly study the effect of QD treatment, CsPbIBr_2_ films were selected for further investigation due to their superior thermal stability and promising bandgap (2.05 eV) for serving as top cells in tandem solar cell applications.^[^ ^52^ ^]^ Many efforts have been dedicated to optimizing the film quality as well as solar cell performance through intermolecular exchange,^[^ ^53^ ^]^ additive introduction^[^ ^20^ ^]^ and interfacial engineering.^[^ ^54^ ^]^ However, the efficiencies of most CsPbIBr_2_ solar cells are below 10% and still far lagged behind the theoretical value (22.1%),^[^ ^55^ ^]^ which is widely recognized to be an urgent issue to solve. Using CsPbIBr_2_ as a prototype, we comprehensively studied the effect of QD treatment to reveal the passivation mechanism and the photophysics and to correlate the treatment to the solar cell performance.

To examine the surface morphology, atomic force microscopy (AFM) measurements were conducted on the films with and without the QD treatment. As shown in Figure S2 in the Supporting Information, the average roughness is 14.9 nm for the QD treated film and 29.6 nm for the control film, which indicates a significantly reduced roughness after the QD treatment. Such an optimized surface morphology of the QD‐treated perovskite films is critical for reducing the current leakage in solar cell devices. Additionally, Figure S3a in the Supporting Information shows the PL spectra of QD‐treated film before and after annealing, as well as the pristine film without QDs. The result indicates that CsPbBr_3_ QDs still remained after spin coating, but the PL peak characteristic of QDs completely disappeared after annealing (see Figure S3b in the Supporting Information), suggesting that the QDs were fully merged into the crystalline film to form a uniform composition. This is also evidenced by the observation of the same PL characteristics in the annealed QD treated CsPbIBr_2_ film excited from either the glass side or the surface side, as shown in Figure S3c in the Supporting Information.

**Figure 3:**
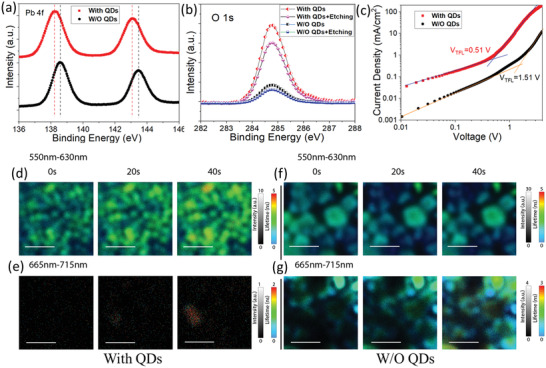
XPS core spectra of a) Pb 4f and b) O 1s in the CsPbIBr_2_ films with and without the QD treatment. c) Dark *J*–*V* curves of the two CsPbIBr_2_ films. PL mapping results for d,e) the QD‐treated CsPbIBr_2_ film and f,g) the control film in the wavelength ranges of 550–630 nm (original phase) and 665–715 nm (segregated phase). The length of the scale bars is 2 µm.

Then, X‐ray photoelectron spectroscopy (XPS) was applied to detect the elemental state and distribution of CsPbIBr_2_ films. As shown in **Figure** **3**a, the binding energy of Pb 4f  in the QD‐treated film shifts towards lower binding energy compared with that in the control film, suggesting the formation of coordination bonds between carboxyl moieties (from OA) and under‐coordinated Pb^2+^ ions.^[^ ^24^ ^]^ To detect the distribution of oxygen element originating from the OA ligands, O 1s signals of both films were collected with and without Ar etching. The samples were etched by Ar ions for 5 min to remove the surface materials, which thereby can give the information of elemental distribution of inner crystalline film.^[^ ^56^, ^57^ ^]^ As shown in Figure 3b, it is clear that the signal intensity of O 1s in the QD‐treated film is much higher than that of the control film, indicating that organic ligands from the QDs are located on the surface. After Ar ion etching, the intensity of O 1s in the QD‐treated film is still much higher than that of the control film, suggesting that organic ligands still exist in the film, most likely at the grain boundaries. It is well recognized that organic ligands locating on the surfaces and grain boundaries can passivate the defects, thus improving the carrier lifetime, as evidenced by the TRPL results shown in Figure 2.^[^ ^33^ ^]^ Halide vacancies as a specific type of point defect in perovskite films are known to be prevalent due to low formation energies, which can promote ion diffusion and lead to device degradation.^[^ ^50^, ^58^ ^]^ Based on the PL and XPS results delineated above, we speculate that the ions from CsPbBr_3_ QDs are released into the thin film to passivate point defects during the annealing, while the hydrophobic ligands of QDs self‐assemble at the surfaces and grain boundaries to enhance the film stability.

To understand the effect of organic ligands from CsPbBr_3_ QDs, CsPbIBr_2_ films were fabricated using hexane with an extremely small amount of OA and OAm (without QDs) as the anti‐solvent. As shown by the SEM image in Figure S4a in the Supporting Information, significantly heterogeneous spots and voids are observed, which are marked with red cycles. Additionally, the carrier lifetime of this film calculated using single‐exponent fitting is 3.92 ns (Figure S4b, Supporting Information), which is comparable to that of the control CsPbIBr_2_ film without QD treatment but much lower than that of QD treated CsPbIBr_2_ film. Therefore, the improved quality of CsPbIBr_2_ treated with QDs is primarily attributed to the QD incorporation. In the later discussion and analysis, only CsPbIBr_2_ films treated with pure hexane and CsPbBr_3_ QDs are included.

Space‐charge‐limited current (SCLC) technique was used to investigate the trap density of both films.^[^ ^59^, ^60^ ^]^ The trap density *n* could be calculated using the equation *n* = 2*ε*_0_ *ε*_*r*_ *V*_TFL_/*qL* ^2^, where *ε*_0_ is the vacuum permittivity, *ε*_r_ is the relative dielectric constant, *V*_TFL_ is trap‐filled limit voltage, *q* is the elementary charge, and *L* is the sample thickness. As shown in Figure 3c, *J*–*V* characteristics of the perovskite films indicate that the trap density is 2.6 × 10^16^ cm^−3^ for the QD‐treated film and 8.9 × 10^16^ cm^−3^ for the control film, evidencing that the trap density in CsPbIBr_2_ film is suppressed after the QD treatment. In addition, we performed Kelvin probe force microscopy (KPFM) measurements under light and dark conditions, where the difference between the contact potential difference (CPD) values under light and dark is noted as the surface photovoltage (SPV).^[^ ^61^, ^62^ ^]^ As shown in Figure S5 in the Supporting Information, compared to dark conditions, the CPD obtained under light is higher for both types of films, which indicates that the light can induce surface dipole accumulation.^[^ ^63^ ^]^ The SPV of the QD‐treated film is 0.12 eV, while it is 0.16 eV for the control film. The lower SPV magnitude in the QD‐treated film suggests a lower degree of dipole accumulation and thus a reduced trap density at the surface.^[^ ^64^, ^65^ ^]^

Light‐induced phase segregation in the mixed‐halide perovskite films was studied using PL measurements and confocal fluorescence microscopy. As shown in Figure S6 in the Supporting Information, the control CsPbIBr_2_ film demonstrates two PL peaks under continuous illumination, one peak located at 585 nm and the other at 675 nm, indicating the occurrence of phase segregation as a result of the formation of I‐rich domains and Br‐rich domains. On the other hand, the CsPbIBr_2_ film after the QD treatment shows only one PL peak at 585 nm under the same condition, which confirmed the suppression effect on phase segregation of the QD treatment. Fluorescence imaging was performed on both films to visualize the phase segregation process under continuous illumination. As shown in Figure 3d–g, two wavelength ranges of PL emission, namely 550–630 nm (intrinsic PL) and 665–715 nm (phase‐segregation induced PL emission), were monitored simultaneously. It is clear that phase segregation is significantly suppressed in the QD‐treated film, while the iodine‐rich PL emission quickly emerges across the whole control film under the same illumination condition. These results indicate that phase segregation was remarkably suppressed in the QD‐treated film but occurred quickly in the control film. The suppressed phase segregation can be attributed to the reduced surface defects and immobile ions due to the passivation effect of the QD treatment.^[^ ^66^, ^67^, ^68^, ^69^, ^70^ ^]^

**Figure 4:**
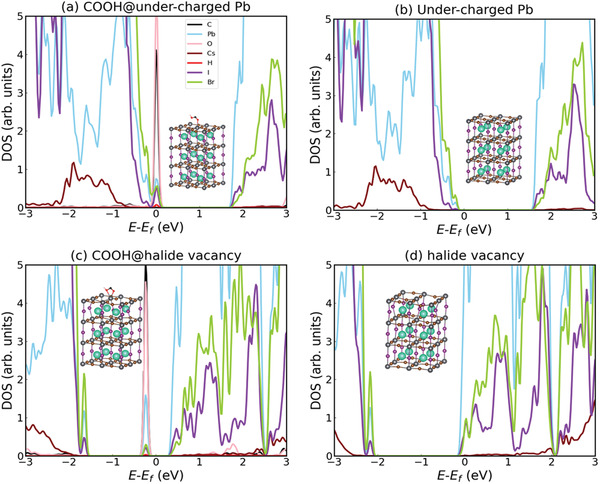
DFT calculated DOS: a) COOH@under‐charged Pb atoms, b) Under‐charged Pb atoms, c) COOH@halide vacancy, and d) halide vacancy systems. Cs, Pb, I, Br, C, O, and H atoms are represented with cyan, grey, purple, brown, black, red, and pink spheres, respectively. Electronic orbitals stemming from the C, H, and Pb atoms notably hybridize around the Fermi energy.

To further understand the effect of CsPbBr_3_ QD treatment on CsPbIBr_2_ films, DFT calculations were carried out with a focus on the passivation of under‐charged Pb^2+^ and halide vacancies with carboxyl (—COOH) groups. The details of the employed computational method are described in the Experimental Section. The fully relaxed COOH@CsPbIBr_2_ geometry shows that the COOH molecule is strongly coordinated with a Pb^2+^ ion on the surface of the halide perovskite slab. In terms of density of states (DOS) (**Figure** **4**a,b), it can be observed the appearance of highly hybridized O, C, and Pb electronic orbitals around the Fermi energy level, which signals the formation of atomic bonds between the carboxyl groups and surface Pb^2+^ ions as a result of charge exchanges between the COOH molecules and halide perovskite surface. When Br vacancies present on the surface of the halide perovskite, similar electronic DOS features are also observed, indicating the interactions between the functional carboxyl group and CsPbIBr_2_ are intensified (Figure 4c,d). Overall, our DFT calculations make plausible the assumption that COOH molecules can successfully passivate under‐charged Pb atoms and halide vacancies on CsPbIBr_2_ surfaces.

To compare their stability against humidity, the CsPbIBr_2_ films were stored in dark under the ambient condition with relative humidity (RH) level of 85%. Figure S7 in the Supporting Information shows the photos of both samples after different durations of storage. The CsPbIBr_2_ film without the QD treatment exhibited fast degradation as the red color completely disappeared within 48 h. In contrast, the appearance of the QD‐treated film remained unchanged for 5 days, and the disappearance of color occurred only after 10 days. Additionally, as shown in Figure S8 in the Supporting Information, water contact angle measurements show that the film with QD treatment has a contact angle of 67 degree while the film without treatment has a contact of 55 degree, which indicates the former film is more hydrophobic, suggesting better moisture resistance. These results strongly support that QD treatment can significantly enhance the moisture stability of perovskite films.

**Figure 5:**
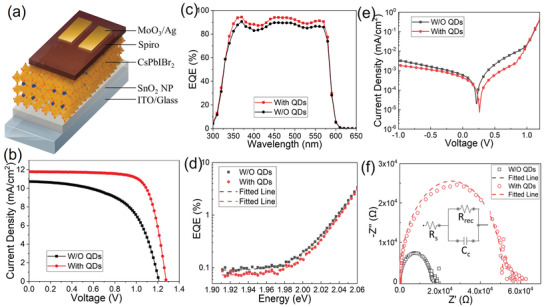
a) Schematical architecture, b) *J*–*V* characteristics, c) EQE curves, and d) the semi‐logarithmic plot to extract the Urbach energy. e) Dark current and f) EIS curves of the CsPbIBr_2_ solar cells.

To examine the impact of different thin films on device performance, two batches of solar cells were fabricated based on the CsPbIBr_2_ films treated with and without CsPbBr_3_ QD solution. **Figure** **5**a shows the schematical device architecture of solar cells, where SnO_2_ nanoparticle layer, CsPbIBr_2_ layer, Spiro (Spiro‐OMeTAD), and MoO_3_/Ag serve as the electron transport layer, light‐absorbing layer, hole transport layer, and anode electrode, respectively. The cross‐sectional SEM images of both solar cells are given in Figure S9 in the Supporting Information, which shows that the thickness of all layers is identical, excluding the effect of thickness on the device performance. However, it is clear that many voids were observed in the cross‐sectional SEM image of the control solar cell, indicating that QD introduction in antisolvent can remarkably improve the quality of perovskite films. Figure 5b shows the *J*–*V* curves of both champion solar cells. The solar cell with QD treatment achieved a PCE of 11.1%, together with an open‐circuit voltage (*V*_oc_) of 1.28 V, a short‐circuit current density (*J*_sc_) of 11.6 mA cm^−2^ and a fill factor (FF) of 75%. In contrast, the control cell delivered a PCE of 8.7% with a *V*_oc_ of 1.22 V, a *J*_sc_ of 10.8 mA cm^−2^, and a FF of 66%. Clearly, all parameters of the solar cell with QD treatment were improved, further confirming that QD treatment is beneficial for solar cell performance. It should be noted that the 11.1% efficiency reported here is among the highest efficiency values for CsPbIBr_2_ solar cells, as shown in Table S1 in the Supporting Information, thus revealing great promise of QD introduction method. Additionally, hysteresis index (HI), which is defined as (PCE_reverse_ − PCE_forward_)/PCE_reverse_, was investigated in both devices under different sweep directions. The calculated HI is 11.5% and 30.0% for the solar cells treated with QDs and without QDs, respectively, as shown in Figure S10 in the Supporting Information. The performance statistics for two batches of solar cells are given in **Table** **2**. To verify the *J*_sc_, external quantum efficiency (EQE) measurements were carried out on both solar cells (Figure 5c). The integrated *J*_sc_ is 11.1 mA cm^−2^ for the solar cell with QD and 10.0 mA cm^−2^ for the control one, which are in good agreement with the *J*–*V* results.

**Table 2:** Statistics for the device performance of CsPbIBr_2_ solar cells (the champion parameters in parenthesis and 24 devices for each type)

Device Type	*V*_OC_ [V]	*J*_SC_ [mA∙cm^−2^]	FF	PCE [%]
With QDs	1.25 ± 0.04 (1.29)	11.4 ± 0.6 (11.6)	0.74 ± 0.02 (0.75)	10.7 ± 0.3 (11.1)
W/O QDs	1.21 ± 0.04 (1.22)	10.7 ± 0.7 (10.8)	0.64 ± 0.03 (0.66)	8.3 ± 0.3 (8.7)

Regarding the influence of energy levels on the device performance, ultra‐violet photoelectron spectrum (UPS) was conducted to investigate the Fermi level and valence band of CsPbIBr_2_ films with and without the QD treatment. Figure S11 a,b in the Supporting Information show the UPS spectra of the cutoff and valence band edge regions. The Fermi level, *ϕ*  =  21.22 − *E*_cutoff_, were confirmed to be −4.10 and −4.81 eV for the QD treated CsPbIBr_2_ film and the control film, respectively. In addition, the valence band maximum, VBM  =  21.22 − (*E*_cutoff_ − E_onset_), were calculated to be −5.86 eV for QD treated CsPbIBr_2_ film and −5.67 eV for the control CsPbIBr_2_ film. Combining with the bandgap, the minimum conduction band were located at −3.81 eV for the QD treated film and −3.63 eV for the control film (Figure S11c, Supporting Information). From the results above, it is clear that the Fermi level of QD treated film is located at the middle of bandgap, which indicates that the QD treated film is more intrinsic than the control one, suggesting a reduced defect density.^[^ ^71^, ^72^ ^]^ This is consistent with the SCLC result. The energy levels of QD treated CsPbIBr_2_ and the control cells are given in Figure S11d,e in the Supporting Information, respectively, where the energy level of transporting layers and electrode materials are extracted from the literature.^[^ ^73^, ^74^ ^]^ Therefore, after QD treatment, the conduction band energy of CsPbIBr_2_ becomes closer to that of the ETL, improving electron transport.^[^ ^75^ ^]^ This is important in CsPbIBr_2_ solar cells because the electron mobility in CsPbIBr_2_ is not as high as hole mobility.^[^ ^76^, ^77^ ^]^

The device stability was also compared by storing both solar cells in ambient condition with RH of 85%. As shown in Figure S12 in the Supporting Information, the control device efficiency completely deteriorated within 24 hours, while the solar cell with QD treatment remained 60% of initial efficiency even after 96 hours, which further confirms that QD treatment can boost device stability. Additionally, different concentrations of QD hexane solution were used to optimize the perovskite films, including 10, 20, and 30 mg mL^−1^ solution. As shown in Table S2 in the Supporting Information, the solar cell without QDs treatment achieved an average efficiency of 8.3%, while all the solar cells with QD treatment delivered higher efficiencies above 10%. The best performing cell is the one treated with 20 mg mL^−1^ QD achieving the highest average efficiency of 10.7%.

Urbach energy is an indicator of energy bandtail to reflect defect density. In general, a smaller Urbach energy implies a lower defect density.^[^ ^78^, ^79^ ^]^ As shown in Figure 5d, the Urbach energy calculated from EQE spectra is 16.94 eV for the solar cell with the QD treatment and 17.45 eV for the control one, indicating notably reduced defect density in QD treated film. Additionally, the junction quality of the devices was also examined by the dark *J*–*V* measurements as shown in Figure 5e. The reverse saturation current is suppressed significantly in the QD treated device, indicating an increased shunt resistance. In the forward bias region at high voltage range > 0.75 V, the dark current for both devices show similar increasing trend, suggesting that the series resistance remained unchanged. This result indicates a better rectifying junction for the solar cell with QDs compared to the control device.^[^ ^80^, ^81^ ^]^ Finally, electrochemical impedance spectroscopy (EIS) measurements were also performed on both solar cells to investigate the quality of interfaces. Figure 5f shows the Nyquist plots of both devices at the open‐circuit voltage bias in dark conditions, and the inset shows the equivalent circuit. The intersection between the spectra and the Z′‐axis at high and low frequencies represents the series resistance (*R*_s_) and recombination resistance (*R*_rec_) of the device, respectively.^[^ ^82^, ^83^, ^84^ ^]^ It is observed that the *R*_s_ of the two types of devices are very close, whereas *R*_rec_ of the device with QD treatment is much larger. The results of unvaried *R*_s_ but increased *R*_rec_ suggest that current leakage or carrier recombination is suppressed due to the introduction of the QD in the anti‐solvent solution, hence improving the device performance. These results are in good agreement with the dark current measurements.

## 3 Conclusion

In this work, we have demonstrated that adding QD in the anti‐solvent solution can reduce trap density, suppress phase segregation and improve film stability for inorganic and hybrid perovskite films. First, as growth seeds, QDs in the anti‐solvent can accelerate the crystallization of perovskite precursor solution, which enables larger grain crystals and improves surface morphology of the resulting films. Second, the ions of CsPbBr_3_ can be released to passivate the vacancy defects during the annealing processes. Meanwhile, carboxyl groups of organic ligands on the QDs can be incorporated and passivate the under‐coordinated Pb^2+^ ions and halide vacancies, further reducing the trap density. Finally, hydrophobic organic ligands can self‐assemble on the surfaces and grain boundaries, which remarkably enhances moisture stability. Based on these findings, CsPbIBr_2_ solar cell achieved an increased PCE from 8.7% to 11.1% as well as enhanced stability after QD addition in the anti‐solvent solution. Overall, this work provides a promising guideline to fabricate high‐quality perovskite films for optoelectronic device applications.

## Conflict of Interest

The authors declare no conflict of interest.

## Supporting information

Supporting Information

## Data Availability

Research data are not shared.
