# Supplementary material for: Quantum Dot Passivation of Halide Perovskite Films with Reduced Defects, Suppressed Phase Segregation, and Enhanced Stability
Source: Adv Sci (Weinh). 2021 Nov 29;9(2):2102258. doi: 10.1002/advs.202102258 (PMC8805552; doi:10.1002/advs.202102258)
Supplement: Supplementary file 1 — Supporting Information [file ADVS-9-2102258-s001.pdf]

WILEY-VCH

ADVANCED SCIENCE
Open Access

Supporting Information

for *Adv. Sci.*, DOI: 10.1002/advs.202102258

Quantum Dot Passivation of Halide Perovskite Films with Reduced Defects, Suppressed Phase Segregation, and Enhanced Stability

Long Hu, Leiping Duan, Yuchen Yao, Weijian Chen, Zizhen Zhou, Claudio Cazorla, Chun-Ho Lin, Xinwei Guan, Xun Geng, Fei Wang, Tao Wan, Shuying Wu, Soshan Cheong, Richard D. Tilley, Shanqin Liu, Jianyu Yuan, Dewei Chu, Tom Wu* and Shujuan Huang**

Supporting Information

Quantum Dot Passivation of Halide Perovskite Films with Reduced Defects, Suppressed Phase Segregation, and Enhanced Stability

Long Hu, Leiping Duan, Yuchen Yao, Weijian Chen, Zizhen Zhou, Claudio Cazorla, Chun-Ho Lin, Xinwei Guan, Xun Geng, Fei Wang, Tao Wan, Shuying Wu, Soshan Cheong, Richard D. Tilley, Shanqin Liu, Jianyu Yuan, Dewei Chu, Tom Wu* and Shujuan Huang**

Methods

Materials preparation

All following chemicals were purchased from Sigma-Aldrich: cesium carbonate (Cs_2CO_3; 99.9%), lead bromide ($PbBr_2$; 99.999%), lead iodide (PbI_2, 99.999%), cesium iodide (CsI, 99.999), MAI (oleic acid (OA; technical grade, 90%), oleylamine (OAm; technical grade, 70%), 1-octadecene (ODE; technical grade, 90%), hexane (reagent grade, >95%), methyl acetate (MeOAc; anhydrous, 99.5%), Spiro-MeOTAD (HPLC, 99%), gold (Au, 99.99%), anhydrous dimethylformamide (DMF, 99.8), anhydrous dimethylsulfoxide (DMSO, 99.9%). Methylamine iodine is from Greatcell Solar (MAI, 99.99%). Tin oxide is from Alfa Aesar (SnO_2, 15% in H_2O colloidal dispersion). All chemicals were used as received without further purification unless mentioned.

$CsPbBr_3$ quantum dot synthesis and purification

0.94 mmol $PbBr_2$, 2.5 ml OA, and 25 ml ODE were loaded in a 100 ml three-neck flask and pumped at 100 ^oC for 30 min. Then, 2.5 mL OAm was injected into the flask. The mixture was completely dissolved to form a clear solution under stirring and heating. The temperature was increased up to 160 °C under N_2 purging, then prepared 2 mL of Cs precursor was swiftly injected into the solution under N_2 purging. The reaction was quenched in an ice bath for 20 s. Then 3-fold volume MeOAc was added in synthesized NC solution, followed by centrifuging at 8000 rpm for 5 min. The supernatant was discarded and the NC pallet was again suspended in hexane/MeOAc (5 mL each) and centrifuged at 7500 rpm for 2 min. The purification process was repeated three times. Afterward, the resultant pellet was dispersed in hexane.

Bulk film deposition

0.6 M $CsPbBr_3$ DMSO precursor solution was prepared by dissolving CsBr and $PbBr_2$ with the molar ratio of 1:1 under stirring and heating at 60 ^oC for 12 h. 1 M $CsPbIBr_2$ and $CsPbBrI_2$ DMSO precursor solutions were prepared by dissolving CsI and $PbBr_2$, and CsBr and PbI_2 into DMSO with the molar ratio of 1:1, respectively, under stirring and heating. 1 M $MAPbI_3$ DMF precursor solution was obtained using MAI and PbI_2 with the molar ratio of 1:1 under the same condition. Before film deposition, all solutions were filtered.

Solar cell fabrication

Patterned ITO glasses were cleaned using detergent, de-ionic water, isopropanol, and acetone, then O_2 plasma was used to treat the ITO surface for 1 min. Diluted SnO_2 solution with a weight concentration of 2.5% was spin-coated on ITO glasses and annealed on a hotplate at 120 ^oC for 30 min in ambient condition. Before halide perovskite deposition, SnO_2 films were treated with O_2 plasma for 1 min. Then the SnO_2 substrates were transferred to a glove box filled with nitrogen. All precursor solutions were spin-coated on ITO/SnO_2 substrate at 1500 rpm for 15 s and then at 4500rpm/45 s. In the spin-coating process of the second step, after 30 s, anti-solvents $CsPbBr_3$ QD hexane solution or pure hexane were dropped rapidly. Then, inorganic perovskite films were annealed at 150 ^oC for 10 min and $MAPbI_3$ film was annealed at 100 ^oC for 10 min.

For device fabrication, 72.3 mg/mL of Spiro-OMeTAD solution in CB, mixed with 28.8 µL t-BP and 17.5 µL Li-TFSI (520 mg mL^{-1}) solution in acetonitrile, was spin-coated on the as-prepared $CsPbIBr_2$ perovskite film at 4000 rpm for 30 s. Finally, 8 nm of MoO_3 and 120 nm of Ag electrode were deposited by thermal evaporation through a shadow mask to form 0.075 cm^2 devices under a vacuum of 2×10^{-6} mbar.

Characterizations

An *I*–*V* tester equipped with a Keithley 2400 source meter was used to measure the current density–voltage (*J*–*V*) under the standard 1 sun illumination from an AM 1.5G solar simulator. An Autolab PGSTAT - 30 w electrochemical impedance spectrometer (EIS) in the glove box was used to investigate the interface properties of $CsPbIBr_2$ solar cells. A frequency analyzer module was adapted in the EIS measurement with a frequency range of 100–106 kHz. The surface morphology was examined by atomic force microscopy (AFM) (Bruker Dimension ICON SPM) with a scan size of “5 µm × 5 µm” and a scan rate of 0.512 Hz. Field emission scanning electron microscopy (SEM), FEI Nova NanoSEM 230 was used to acquire SEM images. TEM images were obtained using a JEOL JEM-2010 and JEOL JEM-F200 operated at 200 kV. Steady-state PL measurements were conducted by WITec Alpha300 confocal Raman spectroscopy to investigate the PL behavior under a 450 nm laser with a fixed light excitation intensity of 1 mW/cm^2. UV–Vis absorption spectra were acquired using a U-4100 spectrophotometer (Hitachi). The PL decay traces and fluorescence lifetime images were measured on a Micro Time 200 (Picoquant) confocal microscope using the TCSPC technique with one-photon excitation of a 470 nm laser. Two identical single-photon avalanche photodiode (APD) detectors were used; therefore, dual-channel signals were recorded simultaneously. All PL measurements were undertaken at room temperature. A silicon tip coated with platinum silicide was used in JEOL JSPM 5400 MkII for Kelvin probe force microscopy (KPFM) measurement, and the work function (WF) of the tip was calibrated by a standard highly oriented pyrolytic graphite (HOPG) sample. XPS and UPS measurements were conducted by a VG ESCALAB MK2 system with monochromatized Al Kα radiation under a pressure of 5.0×10^{-7} Pa. Contact angle measurements were conducted by a Data physics OCA-20 system at room temperature in an ambient atmosphere.

Urbach energy calculation

To investigate the Urbach energy in the absorber, EQE spectrum tails which are also called as "Urbach tail" of the device with high sensitivity have been measured and analyzed. The Urbach energy in the absorber of the device can be estimated by fitting the EQE tail with an exponential growth model as below:[1-3]

$$EQE(E) \propto \alpha(E) \propto \exp(\frac{E}{E_U})$$

where α, E, and E_U are the light-absorbing coefficient, the photon energy, and the estimated Urbach energy, respectively. As the photocurrent response edge of the device is dependent on the perovskite material $CsPbIBr_2$ which owns an optical bandgap of 2.05 eV, any further response lower than 2.05 eV observed in the EQE spectrum is suspected related to the excitation of the charge transfer (CT) states which can indicate the energetic disorder in the active layer.[4, 5]

Computational Details

First-principles calculations based on density functional theory (DFT) [6] were carried out to investigate the mechanism of $CsPbIBr_2$ surface passivation induced by quantum dots (QD) treatment. The Perdew-Burke-Ernzerhof (PBE) version of the generalized gradient approximation (GGA) [7] as implemented in the VASP software [8] was used to describe the ground-state electronic properties of the material. The projector-augmented wave method (PAW) [9] was employed to represent the ionic cores and the following electronic state were considered as valence: Cs 5s 5p and 6s; Pb 6p and 5d; I 4d 5s and 5p; C 2s and 2P; O 2s and 2p; H 1s. The energy cutoff was set to be 520 eV. A 5-atom unit cell of $CsPbIBr_2$ was first relaxed with a Monkhorst-Pack k-point mesh of (12 × 12× 12). Subsequently, a 2 × 2× 6 slab system reproducing the surface Miller index <001> was generated in which the Pb and Br ions were exposed to vacuum. A large enough vacuum layer of 25 Å was introduced in the simulation cell to avoid spurious interactions between periodic images. For the slab calculations, we employed a *k*-point grid of 4 × 4 × 1. Several possible carboxyl absorption geometries were explored by initialising the structural relaxations from different molecular orientations and molecular centre of mass locations. All relaxations were halted when the forces in the atoms were all below 0.01 eV/Å. The structure presenting the lowest energy was further analysed.

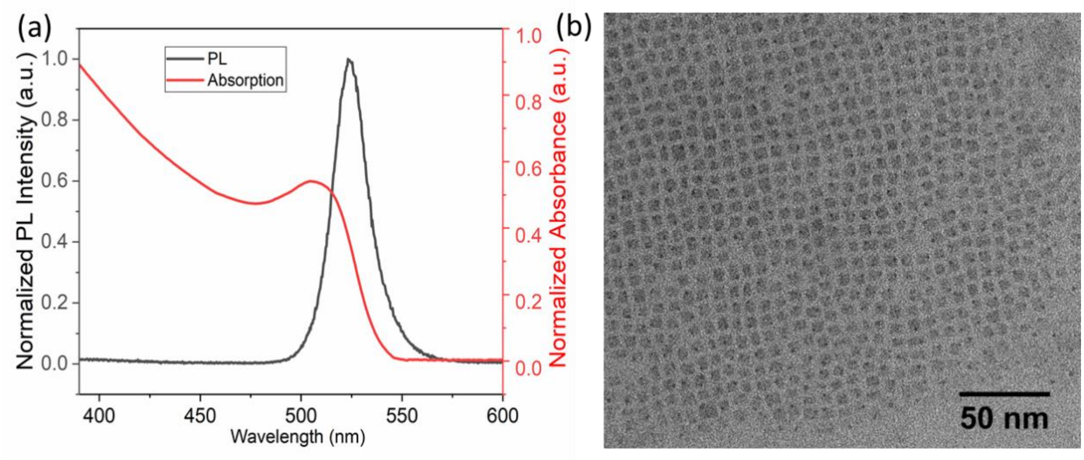

Figure S1. (a) Normalized PL spectrum and UV-vis absorption spectrum of $CsPbBr_3$ QDs. (b) TEM image of $CsPbBr_3$ QDs.

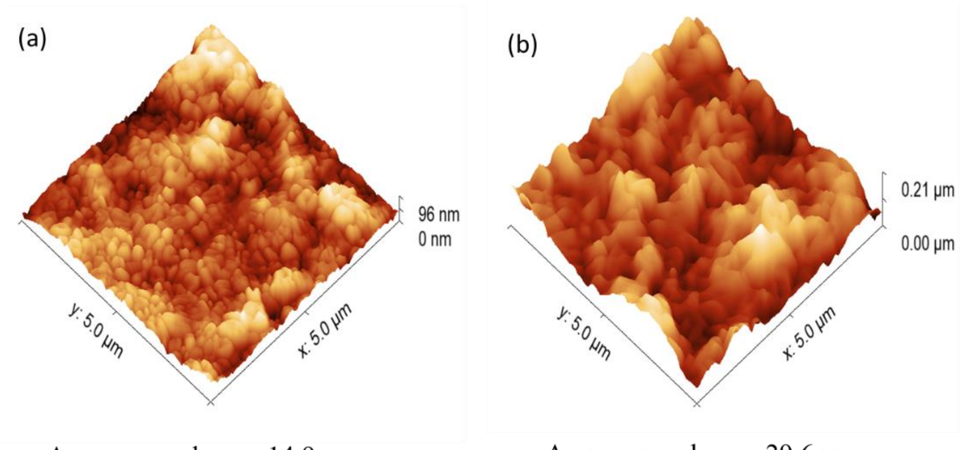

Average roughness: 14.9 nm

Average roughness: 29.6 nm

Figure S2. AFM images of (a) QD-treated $CsPbIBr_2$ film and (b) the control $CsPbIBr_2$ film.

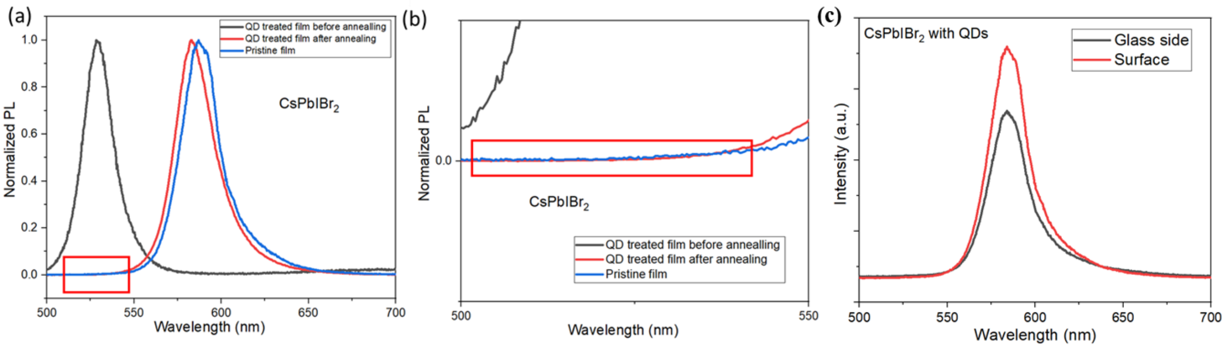

Figure S3. (a) PL spectra of $CsPbBr_3$ QD treated CsPbIBr2 film before and after annealing, and pristine film after annealing, (b) magnified PL spectra. (c) PL spectra of QD treated $CsPbIBr_2$ film excited from glass side and surface side.

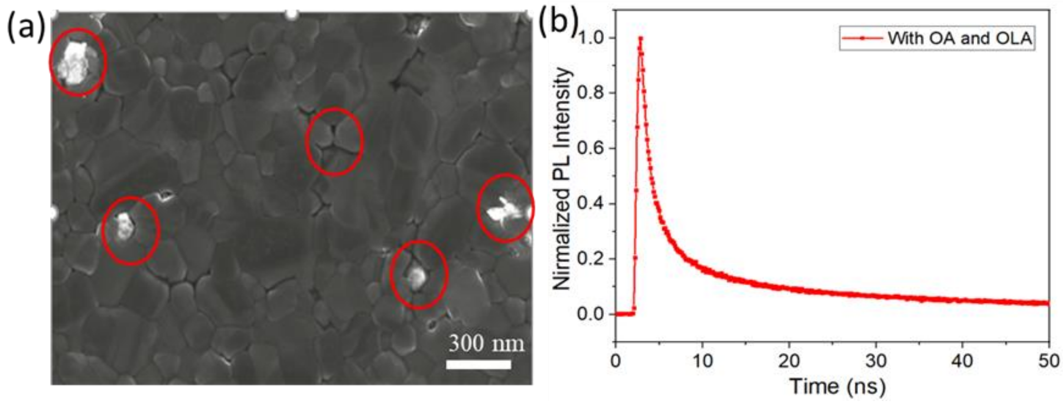

Figure S4. (a) SEM image and (b) TRPL decay curve of $CsPbIBr_2$ film fabricated with OA and OLA in hexane anti-solvent.

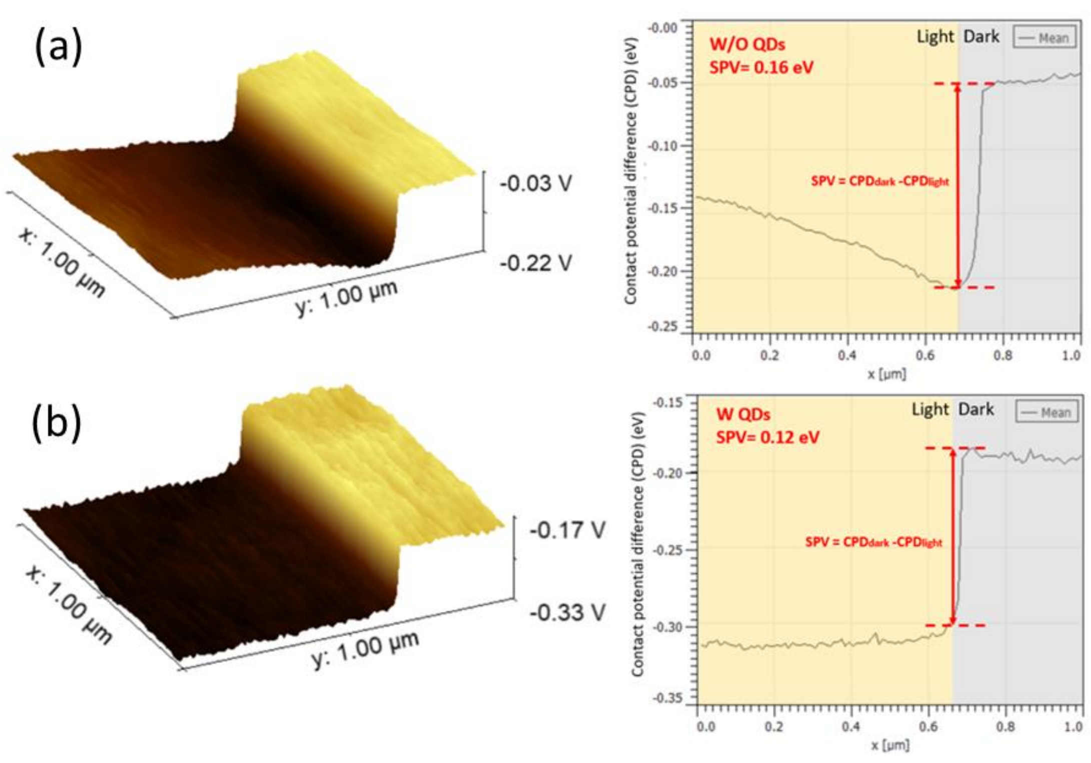

Figure S5. 3D KPFM images measured under light and dark conditions for (a) the control $CsPbIBr_2$ film and (b) QD-treated $CsPbIBr_2$ film with estimated surface photovoltage (SPV).

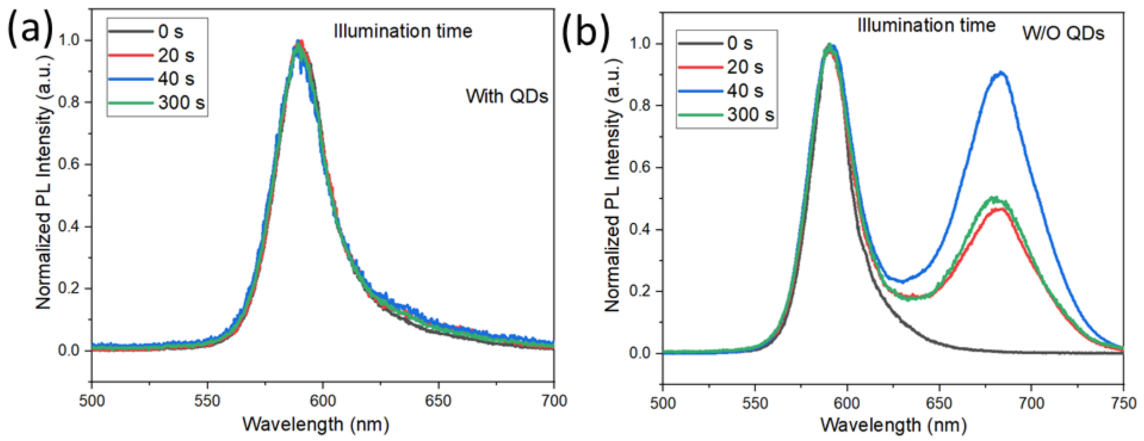

Figure S6. PL spectra of (a) the QD-treated $CsPbIBr_2$ film and (b) the control $CsPbIBr_2$ film under different duration time (0-300 seconds) of the contineous illumination.

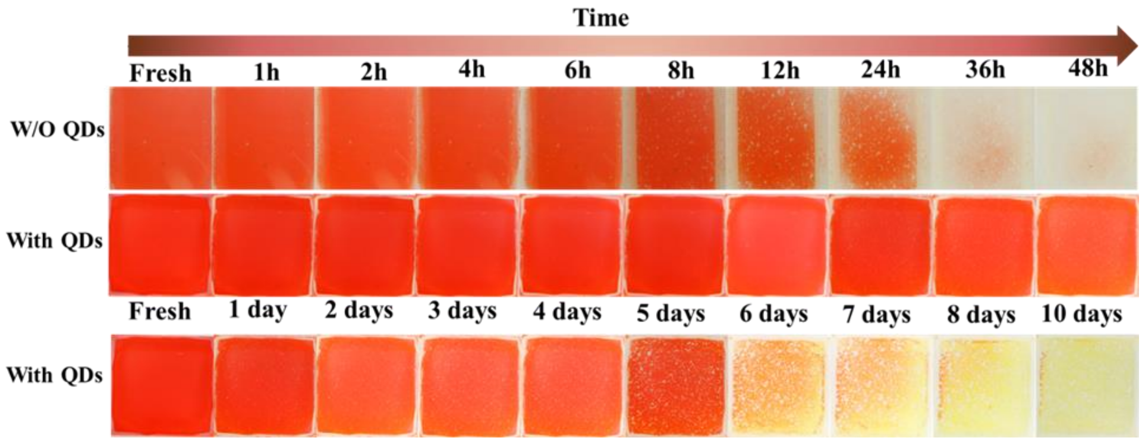

Figure S7. Photographs of $CsPbIBr_2$ perovskite film on glass substrate over time without encapsulation. The samples were stored in an environmental chamber with an RH of 85%.

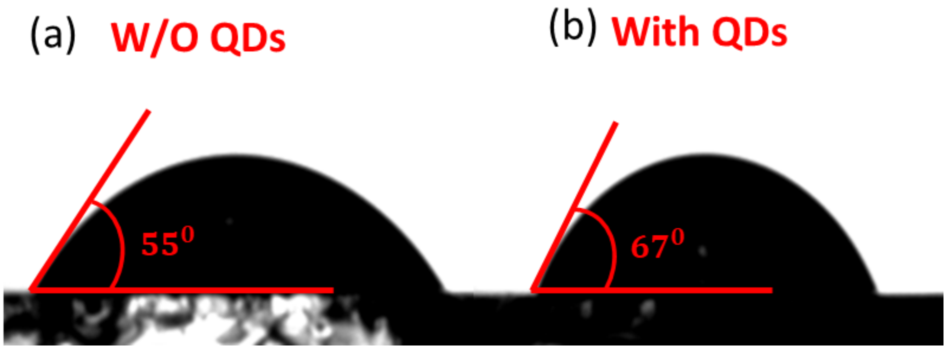

Figure S8. Water contact angles of (a) the control $CsPbIBr_2$ and (b) QD treated $CsPbIBr_2$ films.

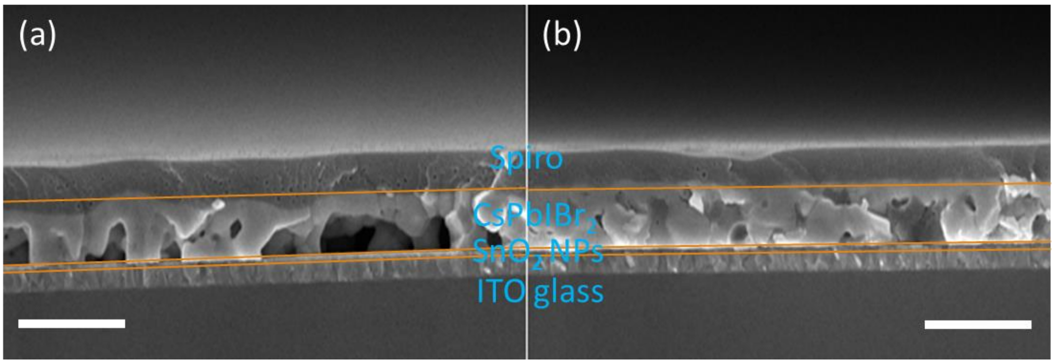

Figure S9. Cross-sectional SEM of (a) the control $CsPbIBr_2$ solar cell (b) QD treated $CsPbIBr_2$ solar cell without MoO_3/Ag layers (scale bar: 300 nm).

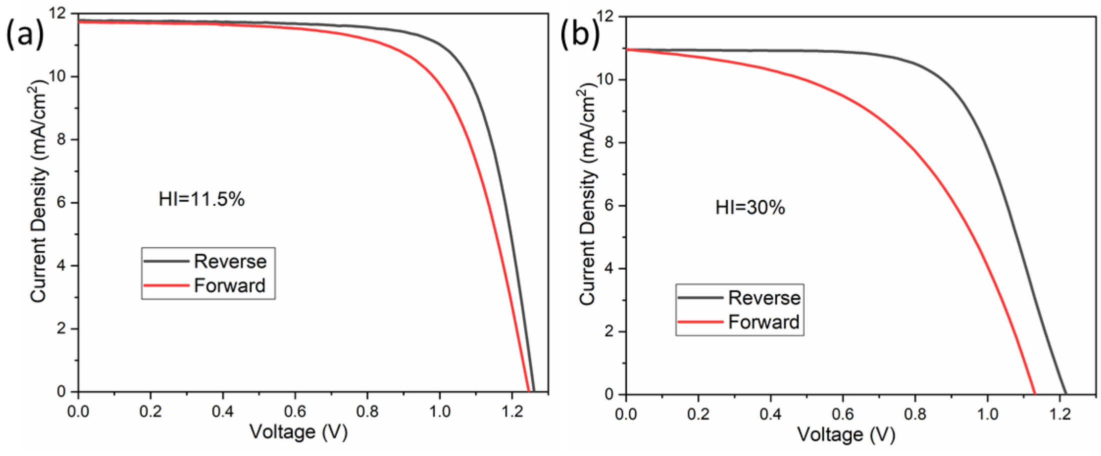

Figure S10. J-V curves of devices (a) of $CsPbIBr_2$ with QD treatment and (b) without QD treatment under different sweep directions.

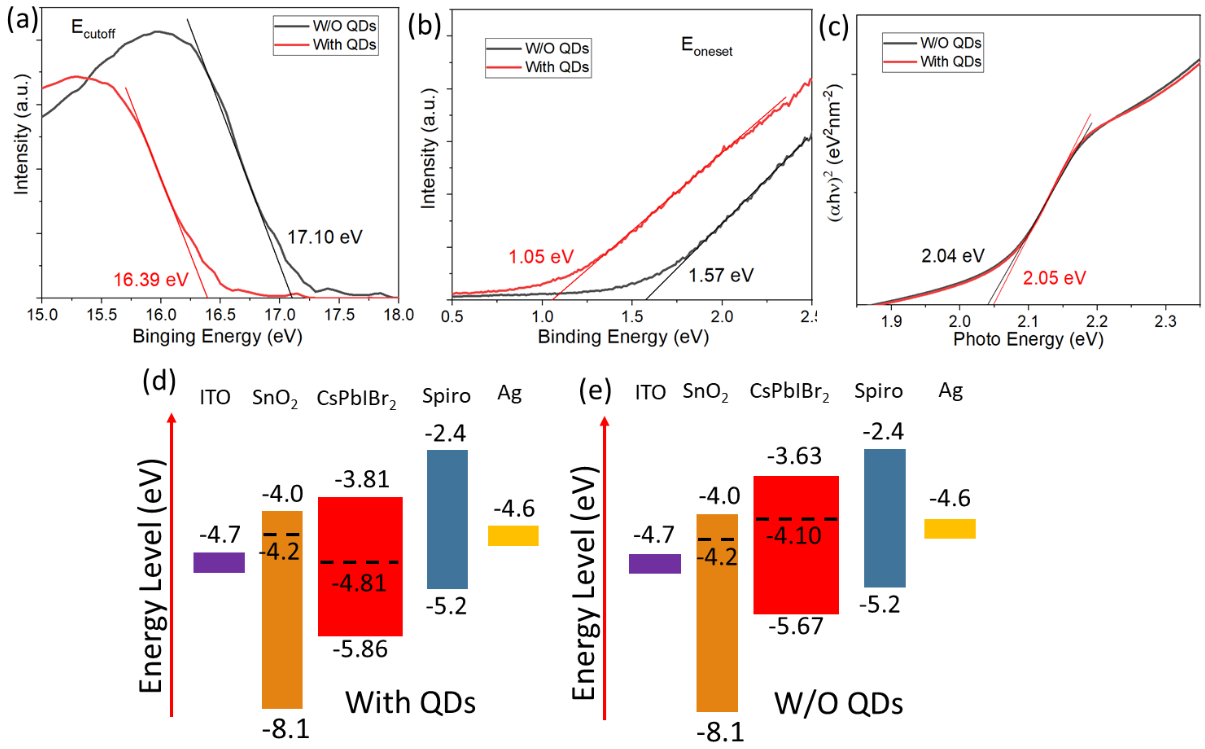

Figure S11. The UPS spectra of the (a) cut-off and (b) valence band edge regions. (c) The bandgap of both films extracted from the absorption data. The energy levels of devices with (d) the QD treated film and (e) the control film.

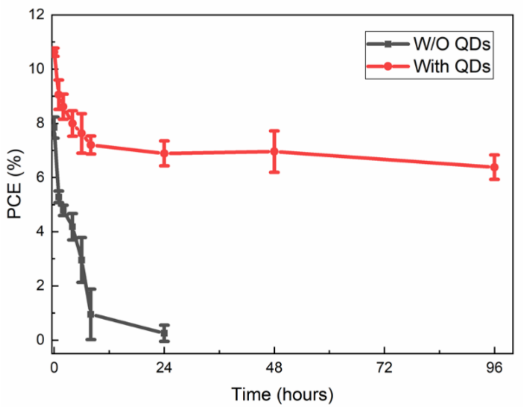

Figure S12. Stability test results of the $CsPbIBr_2$ devices under the ambient condition with an RH of 85%.

Table S1. The reported efficiencies and device structures of $CsPbIBr_2$ solar cells.

Year	Device Structure	Voc (V)	Jsc (mA/cm^2)	FF	PCE (%)	Ref
2016	FTO/c-TiO_2/$CsPbIBr_2$/Au	0.96	8.7	0.56	4.7	[10]
2016	FTO/c-TiO_2/m-TiO_2/$CsPbIBr_2$/Spiro-OMeTAD/Au	1.13	7.8	0.72	6.3	[11]
2017	FTO/NiOx/$CsPbIBr_2$/MoOx/Au	0.85	10.6	0.62	5.5	[12]
2017	FTO/c-TiO_2/$CsPbIBr_2$/Spiro-OMeTAD/Au	1.23	9.7	0.67	8.0	[13]
2017	ITO/SnO_2/C60/$CsPbIBr_2$/Spiro-OMeTAD/Au	1.18	8.3	0.75	7.3	[14]
2017	FTO/c-TiO_2/m-TiO_2/$CsPbIBr_2$/carbon	1.08	12.3	0.62	8.3	[15]
2018	FTO/c-TiO_2/$CsPbIBr_2$/carbon	1.25	10.7	0.69	9.2	[16]
2018	FTO/c-TiO_2/m-TiO_2/$CsPbIBr_2$/carbon	0.96	12.2	0.53	6.1	[17]
2018	FTO/NiOx/$CsPbIBr_2$/ZnO/Al	1.01	8.7	0.64	5.6	[18]
2019	ITO/In_2S_3/$CsPbIBr_2$/Spiro-OMeTAD/Au	1.09	7.8	0.66	5.6	[19]
2019	ITO/SnO_2/$CsPbIBr_2$/carbon	1.23	8.5	0.67	7.0	[20]
2019	FTO/TiO_2/$SmBr_3$/$CsPbIBr_2$/Spiro-OMeTAD/Au	1.17	12.8	0.73	10.9	[21]
2020	FTO/c-TiO_2/PEAI/$CsPbIBr_2$/Carbon	1.34	11.7	0.65	10.2	[22]
2020	FTO/c-TiO_2/SAS-$CsPbIBr_2$/Spiro-OMeTAD/Au	1.21	12.3	0.71	10.6	[23]
2020	FTO/SnO_2/$CsPbIBr_2$/Spiro- OMeTAD/Au	1.24	12.0	0.75	11.1	[24]
2020	FTO/SnO_2/$CsPbIBr_2$/Spiro-OMeTAD/Au	1.27	11.9	0.72	10.8	[25]

2021	ITO/SnO_2/$CsPbIBr_2$/Spiro-OMeTAD/MoO_3/Au	1.29	11.6	0.75	11.1	This work

Table S2. Statistics for device performance for $CsPbIBr_2$ solar cells fabricated with different QD concentration. (The champion parameters in parenthesis and 24 samples for each type)

Device Type	V_{OC} (V)	J_{SC} (mA·cm^{-2})	FF	PCE (%)
W/O QD	1.21 ± 0.04 (1.22)	10.7 ± 0.7 (10.8)	0.64 ± 0.03 (0.66)	8.3 ± 0.3 (8.7)
10 mg/ml	1.24 ± 0.04 (1.27)	11.4 ± 0.7 (11.4)	0.73 ± 0.03 (0.74)	10.3 ± 0.3 (10.7)
20 mg/ml	1.25 ± 0.04 (1.29)	11.5 ± 0.6 (11.7)	0.74 ± 0.03 (0.75)	10.7 ± 0.3 (11.1)
30 mg/ml	1.22 ± 0.04 (1.24)	11.8 ± 0.6 (11.9)	0.72± 0.04 (0.73)	10.4± 0.3 (10.8)

References

[1] W. Gong, M. A. Faist, N. J. Ekins-Daukes, Z. Xu, D. D. C. Bradley, J. Nelson, T. Kirchartz, *Phys. Rev. B* **2012**, 86, 024201.
[2] J. Lee, K. Vandewal, S. R. Yost, M. E. Bahlke, L. Goris, M. A. Baldo, J. V. Manca, T. Van Voorhis, *J. Am. Chem. Soc.* **2010**, 132, 11878.
[3] R. A. Street, K. W. Song, J. E. Northrup, S. Cowan, *Phys. Rev. B* **2011**, 83, 165207.
[4] N. Jain, N. Chandrasekaran, A. Sadhanala, R. H. Friend, C. R. McNeill, D. Kabra, *J. Mater. Chem. A* **2017**, 5, 24749.
[5] H. Yao, Y. Cui, R. Yu, B. Gao, H. Zhang, J. Hou, *Angew. Chem., Int. Ed.* **2017**, 56, 3045.
[6] C. Cazorla, J. Boronat, *Rev. Mod. Phys.* **2017**, 89, 035003.
[7] J. P. Perdew, K. Burke, M. Ernzerhof, *Phys. Rev. Lett.* **1996**, 77, 3865.
[8] G. Kresse, J. Furthmüller, *Phys. Rev. B* **1996**, 54, 11169.
[9] P. E. Blöchl, *Phys. Rev. B* **1994**, 50, 17953.
[10] Q. Ma, S. Huang, X. Wen, M. A. Green, A. W. Ho-Baillie, *Adv. Energy Mater.* **2016**, 6, 1502202.
[11] C. F. J. Lau, X. Deng, Q. Ma, J. Zheng, J. S. Yun, M. A. Green, S. Huang, A. W. Y. Ho-Baillie, *ACS Energy Lett.* **2016**, 1, 573.
[12] C. Liu, W. Li, J. Chen, J. Fan, Y. Mai, R. E. J. N. E. Schropp, *Nano Energy* **2017**, 41, 75.
[13] W. Li, M. U. Rothmann, A. Liu, Z. Wang, Y. Zhang, A. R. Pascoe, J. Lu, L. Jiang, Y. Chen, F. Huang, *Adv. Energy Mater.* **2017**, 7, 1700946.
[14] F. Liu, C. Ding, Y. Zhang, T. S. Ripolles, T. Kamisaka, T. Toyoda, S. Hayase, T. Minemoto, K. Yoshino, S. Dai, *J. Am. Chem. Soc.* **2017**, 139, 16708.
[15] J. Liang, P. Zhao, C. Wang, Y. Wang, Y. Hu, G. Zhu, L. Ma, J. Liu, Z. Jin, *J. Am. Chem. Soc.* **2017**, 139, 14009.
[16] W. Zhu, Q. Zhang, D. Chen, Z. Zhang, Z. Lin, J. Chang, J. Zhang, C. Zhang, Y. Hao, *Adv. Energy Mater.* **2018**, 8, 1802080.
[17] J. Liang, Z. Liu, L. Qiu, Z. Hawash, L. Meng, Z. Wu, Y. Jiang, L. K. Ono, Y. Qi, *Adv. Energy Mater.* **2018**, 8, 1800504.
[18] J. Lin, M. Lai, L. Dou, C. S. Kley, H. Chen, F. Peng, J. Sun, D. Lu, S. A. Hawks, C. Xie, F. Cui, A. P. Alivisatos, D. T. Limmer, P. Yang, *Nat. Mater.* **2018**, 17, 261.
[19] B. Yang, M. Wang, X. Hu, T. Zhou, Z. Zang, *Nano Energy* **2019**, 57, 718.

[20] Z. Guo, S. Teo, Z. Xu, C. Zhang, Y. Kamata, S. Hayase, T. Ma, *J. Mater. Chem. A* **2019**, 7, 1227.
[21] W. S. Subhani, K. Wang, M. Du, X. Wang, S. Liu, *Adv. Energy Mater.* **2019**, 9, 1803785.
[22] W. Zhu, Z. Zhang, D. Chen, W. Chai, D. Chen, J. Zhang, C. Zhang, Y. Hao, *Nano-Micro Letters* **2020**, 12, 87.
[23] Y. Wang, K. Wang, W. S. Subhani, C. Zhang, X. Jiang, S. Wang, H. Bao, L. Liu, L. Wan, S. Liu, *Small* **2020**, 16, 1907283.
[24] W. Zhang, J. Xiong, J. Li, W. A. Daoud, *Small* **2020**, 16, 2001535.
[25] S. Cao, H. Wang, H. Li, J. Chen, Z. Zang, *Chemical Engineering Journal* **2020**, 394, 124903.
